# A review of stingless bees' bioactivity in different parts of the world

**DOI:** 10.25122/jml-2022-0160

**Published:** 2023-01

**Authors:** Lucky Poh Wah Goh, Roslina Jawan, Ainol Azifa Mohd Faik, Jualang Azlan Gansau

**Affiliations:** 1 Biotechnology Programme, Faculty of Science and Natural Resources, Universiti Malaysia Sabah, Kota Kinabalu, Sabah, Malaysia

**Keywords:** stingless bees, kelulut, honey, propolis, antimicrobial, bioactivity

## Abstract

Stingless bees, also known as meliponines, live in beehives. However, reports on the distribution of stingless bees are scattered, resulting in a lack of precision. Honey and propolis are the main components that can be harvested from their beehive, with a great commercial value of up to 610 million USD. Despite the enormous potential profits, discrepancies in their bioactivities have been observed worldwide, leading to a lack of confidence. Therefore, this review provided oversight on the potential of stingless bee products and highlighted the differences between stingless bees in Asia, Australia, Africa, and America. The bioactivity of stingless bee products is diverse and exhibits great potential as an antimicrobial agent or in various diseases such as diabetes, cardiovascular disease, cancers, and oral problems.

## INTRODUCTION

The tropics and subtropics are home to thousands of unique bee species. Meliponini, also known as stingless bees, is one of the species that captured the attention of researchers [1]. Stingless bees have the most diverse species of corbiculate eusocial bees (*Hymenoptera, Apidae: Meliponini*), comprising 60 different groups divided into 600 different species and subspecies [2]. The colony size of stingless bees also varies greatly, with some colonies containing only a few dozen and others containing thousands. Stingless bees play an important role in the pollination process, just like their *Apini* relatives, especially *Apis mellifera*) [3]. The high pollination rate contributes to increased plant biodiversity.

Stingless bee products such as propolis and honey bring high commercial value. For example, it was estimated that the global commercial market for propolis was worth approximately 610 million USD in 2020, with an estimated annual net growth rate of 5.48% from 2021 until 2026 [4]. *A. mellifera* propolis was estimated to constitute the vast bulk of the market for propolis [5].

Honey produced by stingless bees (*Hymenoptera, Apidae: Meliponini*) has been employed in traditional medicine in Peru for at least a few centuries. This honey is collected by felling trees that contain bee colonies, locating the honey pots, and then leaving the nests exposed rather than harvesting them for meliponiculture. Additionally, colonies are commonly found in the walls of homes and other manufactured constructions [6]. Honey is often extracted from honey pots with bare hands, and the honey obtained this way frequently contains pollen and brood. Honey is widely used and frequently sold at local markets in Amazonian Peru, both as a sweetener and, more frequently, as a component in folk medicine, as it is in Guatemala, Mexico, and Venezuela [7].

Honey is a multifaceted substance that contains edaphic, botanical, and entomological components, all of which contribute to the honey's bioactive properties and utility in apitherapy [8]. Several main attributes of honey led to its discovery as a traditional remedy. Firstly, honey has high osmolarity, which causes fluid to be drawn out of tissues and produces a moist environment conducive to healing. Secondly, hydrogen peroxide is produced as a by-product of honey glucose oxidase in action, which contributes to its bioactivity. Finally, it carries antioxidant characteristics and contains properties against bacteria and inflammation [9].

It could be argued that stingless bees significantly exhibit greater biological activities due to the sheer number of species and their extensive geographical distribution across the tropical rainforests of four continents Asia, Australia, Africa, and America, where the majority of the species are still a mystery to solve [10]. Therefore, stingless bees hold a great source of potential which can be further investigated.

## THE BIOLOGICAL POTENTIAL OF PROPOLIS FROM STINGLESS BEES IN ASIA, AUSTRALIA, AFRICA, AND AMERICA

### Asia

*Apis mellifera* and other stingless bee species originated from the south and east of Asia as well as the west. *Heterotrigona itama, Geniotrigona thoracic, Tetragonula laeviceps, Tetragonula iridipennis, Tetragonula biroi, Tetragonula sapiens, Lepidotrigona terminate, Pariotrigona pendleburyi*, and *Lisotrigona furva* were among the species discovered in this region [11, 12]. T. incisa propolis was used to isolate and purify cardol (5-pentadecylresorcinol). Cardol was found to be cytotoxic against SW620 human colorectal cancer cell line [13]. The isolated Cardol from propolis induces apoptosis or programmed cell death and cell arrest in the G0/G1 phase [14, 15]. G0/G1 phase is a cell cycle checkpoint that undergoes B-dependent homologous recombination upon DNA damage [16].

The chemical contents of the two species (*H. Itama* and *G. thoracica*) are very different [17]. A study reported that propolis extracts produced by stingless bees have antibacterial activity against pathogens, with *H. Itama* propolis being more effective than *G. thoracica*. It was reported that the higher phenolic concentration of *H. Itama* propolis was responsible for its antibacterial activity [17]. Furthermore, terpenoids isolated from propolis of Malaysia *H. Itama* bees had anti-inflammatory and antioxidant properties [18, 19]. This indicated that the propolis of *H. Itama* in Malaysia has great beneficial effects.

Additionally, there have been reports of biological activity in a few different preparations of Thai stingless bee propolis described as follows. The Thai stingless bee *T. laeviceps* produces propolis that is cytotoxic to several different cancer cell lines, including BT474, Chago, Hep-G2, KATO-III, and SW620 [20]. Propolis from Thai *T. laeviceps* and *T. melanoleuca* exhibited antibacterial action with triterpenes as the main bioactive compound [21]. However, *T. laeviceps* have higher antibacterial properties as compared to *T. melanoleuca*, which was because of α-Mangostin (α-MG). α-MG is a plant-derived compound with multiple pharmaceutical properties, including antibacterial ones [22, 23]. α-MG causes the breakdown of the cellular membrane of methicillin-resistant *S. aureus* (MRSA), and Gram-positive pathogens do not develop resistance against α-MG [24, 25].

Propolis from two different species of stingless bees, *T. laeviceps* and *T. melanoleuca*, were tested for their ability to inhibit the growth of the pathogen *Cryptococcus neoformans* [26]. Propolis extracts from different species of stingless bees can inhibit the growth of *C. neoformans* from producing chitin and chitosan, both components of the cell wall, as well as melanin, which is a virulence factor [27]. Additionally, the expression of genes such as *CDA1, IPC1-PKC1*, and *LAC1* that are implicated in the cryptococcal melanization pathway was reduced by propolis treatment [26]. The propolis obtained from stingless bees in Vietnam exhibit various biological functions such as anticancer and antimicrobial activities [28–33].

### Australia and Africa

In Australia, little research has been done on the bioactive compounds of propolis produced by stingless bees. Despite this, studies have revealed that the propolis produced by Australian stingless bees has therapeutic properties [34]. For example, the propolis from the Australian stingless bees *T. carbonaria* were reported to exhibit anti-inflammatory activities. The most frequent compounds, according to the gas chromatographic data, were a combination of phenolics and terpenoids, notably pimaric acid, isopimaric acid, and gallic acid [35].

Propolis was reported to have a dose-dependent relaxing effect on the arteries of smooth muscles. Propolis extract of stingless bees also showed antibacterial action against *S. aureus* and was thought to be responsible for therapeutic advantages [36]. Consequently, little research has been done on the bioactivities of the propolis made by African stingless bees. To our knowledge, only two studies have been published in the last ten years that examine the biological activity of African stingless bees. Labdane and abietane diterpenic acids, as well as triterpenes, were found in propolis from the African stingless bee (*M. ferruginae*) [37]. Additionally, propolis extract from the stingless bee *Dactylurina Staudinger* exhibits antibacterial activity for Gram-positive and Gram-negative bacteria infections [38]. Despite the lack of studies, the reports on stingless bees in these regions carry antimicrobial properties.

### America

There is a strong argument that Brazil has conducted the most comprehensive research on the biological activity of stingless bees' antibacterial, anti-inflammatory, and antioxidant biological properties. These arguments were consistent with Asian-origin stingless bees and stingless bee propolis of Australian origin [39]. Propolis compounds from Brazilian stingless bees were found to have antibacterial action against several *Candida* species [40].

American stingless bees propolis contain antioxidant activities, and these properties were observed in different bee species such as *Scaptotrigona* aff. *postica* and *M. subnitida*. The antioxidant activity had several properties, such as radical scavenging, reducing power, and metal-chelating abilities [41]. Propolis extracts stopped cancer cells from migrating, stopped the cell cycle, and triggered apoptosis [42]. Propolis extracts were much less harmful to non-cancerous cells than cancerous ones, highlighting the potential cytotoxicity of propolis extract using mice-fed diets with up to 4000 mg/kg1 of body weight [43]. The anti-inflammatory properties of *Scaptotrigona* aff. *postica* propolis extract had antagonistic effects on asthma [43]. Propolis from *M. subnitida* improved healing lesions' evolution, boosted angiogenesis and collagen synthesis, and decreased inflammatory cell recruitment [35, 44–53]. The research was conducted on rats given propolis extracts, which also effectively suppress the cough brought on by ammonia. Furthermore, there was no evidence of acute toxicity at dosages ranging from 2,000 to 5,000 mg/kg of body weight [54–56]. This highlighted that propolis from *M. subnitida* is one of the major potential products to be utilized in future treatments.

## MELIPONICULTURE

Apiculture or beekeeping using *Apis mellifera* remain a challenge because of the rising threat of various diseases and the increased use of agrochemicals to combat foreign bodies [57]. Meliponiculture focuses on stingless bees and has been widely used, being an attractive element of indigenous cultures in many locations. Due to various circumstances, the slow and catastrophic fall of meliponiculture has been inescapable since the 16^th^ century [58, 59]. Events such as the accidental release of *A. m. scutellata* cause the spread of Africanized population, which affects the *A. mellifera* European lineages [59]. The changes in bee population affected the honey yield in Meliponiculture.

## PROPOLIS

Since the beginning of recorded history, propolis has been utilized as a medicinal ingredient. Propolis is made of a resinous material that can be obtained from many different plants. Propolis has been used to construct hives and protect their colonies. In addition, the propolis produced by stingless bees is referred to as geopropolis because it is produced from plant resins mixed with pollen, wax, salivary secretions, and soil. However, the name "geopropolis" is sometimes used to refer to propolis collected by Meliponini in general, which is not accurate because only some species of Meliponini add soil and clay to the plant resins collected in their nests [60].

Propolis has a high level of biological activity and is associated with a wide variety of beneficial effects on human health, including antiviral, antibacterial, antifungal, anticancer, immunomodulatory, and anti-inflammatory [61]. There is a significant amount of variation in the chemical composition of propolis across different geographical and climatic locales. The area, source of resin, and bee species all play a role in determining the chemical composition of propolis, which in turn results in a wide range of biological activities. In addition to providing physical protection, bee colonies also benefit from the physiological functions and social immunity provided by propolis. Propolis has been reported to possess antimicrobial properties that protect against bee illnesses. Borba and Spivak (2017) reported that propolis conferred a certain degree of protection against the pathogen that causes American foulbrood disease [62]. It has also been established that propolis possesses antiviral properties, specifically the D-Wing viral parasite embodied by the mites. In addition, there is evidence that propolis contributes to the formation of social immunity as well as the management of immunological processes within the hive. Immunologically significant hymenoptaecin and *AmEater* gene expression are repressed by propolis [63, 64]. The overexpression of these genes in bees has serious aftershocks, including a reduction in the bees' longevity, retardation in their capacity for learning, and an overall reduction in the colonies' level of productiveness.

Humans have utilized propolis for around the same amount of time as honey. Surprisingly, the medical application of propolis developed independently throughout millennia in a variety of different ancient communities. According to historical texts, propolis was utilized by ancient peoples in Egypt, Greece, Persia, and Rome to treat a wide variety of illnesses and conditions. It is reported that Hippocrates employed propolis to treat ulcers as well as internal and external wounds of the body. Propolis has been used as a form of traditional medicine for a very long time in India as ayurvedic medicine. The efficacy of propolis as a therapeutic agent has been demonstrated unequivocally and comprehensively by recent studies [65]. Evidence can range from research conducted *in vitro* to human clinical trials that are randomized, controlled, and monitored with placebos [66]. Diabetes, cardiovascular illness, chronic renal disease, cancers, and oral problems all benefit from propolis. It has also been shown that propolis can lessen the intensity of symptoms brought on by acute infections. Most notably, several randomized controlled trials that included a placebo as a comparison group has demonstrated that propolis offers a therapeutic advantage to patients with COVID-19 [67–69].

## ANTIMICROBIAL ROLE OF STINGLESS BEE HONEY

In every wound, there is a population of microorganisms; nevertheless, the vast majority of these organisms do not cause an infection, and the wound eventually heals. However, under certain conditions, the wound can be severely infected. This is due to the weakened immune system that leads to the multiplication of pathogens and transmission of the infection to the host's tissues, which may result in granuloma or an abscess [70]. If this situation continues, it may deteriorate, eventually leading to a disease affecting the entire body. Therefore, an intervention should be carried out as a preventative step as soon as possible. The nursing approach to wound healing emphasizes the elimination of foreign invaders and the prevention of pathogen colonization, both of which are responsible for stalling the wound-healing process.

As reviewed earlier, honey or cerumen contains a large number of microorganisms. When honey is applied to a wound, the effect that this may have on the body's natural ability to heal is a topic of discussion [71]. Most of these bacteria belong to the non-pathogenic *Bacillus* genus, *Actinomycete*, or *Streptomyces*, which do not obstruct the body's natural healing process.

## RESPONSE OF STINGLESS BEE HONEY AGAINST MICROBIAL INFECTIONS

The prevention or treatment of infections, particularly when the patient is healing, is the major objective of antimicrobial treatments. Honey produced by stingless bees can be utilized in pharmaceutical preparations as an antibacterial component because it possesses antimicrobial and antiseptic properties [72].

Components containing peroxide and those that do not contain peroxide are responsible for the antibacterial action of honey. The activity of hydrogen peroxide provides the foundation for the peroxide component, controlled by glucose oxidase and catalase, both necessary enzymes in the honey. Catalase neutralizes the hydrogen peroxide produced by glucose oxidase, which allows the honey's nutritional content to be preserved. Glucose oxidase stimulates the production of hydrogen peroxide. Hydrogen peroxide increases cytokine production, which plays a role in the inflammatory response against germs [73]. The antibacterial and antifungal properties of honey are a result of phytochemical components, such as flavonoids, phenolic compounds, and antibacterial peptides. These components may exert their effects by directly inhibiting phagocytosis, reducing tissue damage caused by superoxide free radicals. The honey's natural acidity, produced by organic acids, is the component that does not contain peroxide. These acids, which make up around 0.57 percent of the honey, will kill the majority of bacteria that thrive at pH levels ranging from 7.2 to 7.4. It is quite unusual to identify non-peroxide activity in honey produced by *A. mellifera*, but Emi *et al*. discovered it in a variety of honey samples produced by stingless bees from different parts of the world [74]. Stow *et al*. conducted an entomological study and found that the cuticular antimicrobial compounds emitted by stingless bee honey were also responsible for inhibiting microbial growth [75]. Honey produced by stingless bees possesses good antibacterial properties; therefore, applying it to an injured region may lower the risk of microbial infection and, as a result, speed up the process of healing [76].

## HARVESTING AND CHARACTERIZATION OF STINGLESS BEE PRODUCTS

The percentages of stingless bee-based elements in the beehive are currently unknown, although these percentages fluctuate based on the bee species. Because the hive itself is constructed out of propolis, the majority of a hive inhabited by stingless bees will contain propolis as its primary component. In a study that looked at nine different species of *Trigona*, the researchers found that the hive consists of 63.7 percent propolis, 20.9 percent beebread, and 15.4 percent honey [77, 78]. In addition, it is uncertain what percentage of each product produced by stingless bees is utilized in the pharmaceutical or commercial industries.

Stingless bees are responsible for the production of honey, which is then stored in the hive in tiny resin pots. In earlier times, honey would typically be extracted from honey pots by pressing them. This approach had several downsides, such as causing pot damage and reducing the number of bees produced. When the pot honey was squeezed, it produced stingless bee honey contaminated with bee bread containing microorganisms. This stimulated the honey to ferment, which produced a variety of honey products such as honey wine and honey vinegar by several microorganisms such as *Saccharomyces cerevisiae, Acetobacter spp*., or *Pediococcus spp*. [79–81]. Therefore, the collection and storage of honey depend on the application of the fermented products.

The chemical makeup of goods derived from stingless bees differs depending on their botanical and geographical origin, the species of bees used, and the local climate. This is true even when harvesting methods and post-harvest management of stingless bee honey have been improved. Hence, there is currently no internationally accepted characterization standard for stingless bee products [69, 82].

## CONCLUSION

This review article shows that stingless bee products have the potential to act as antimicrobial agents in the future. The present article observed that different regions of the world have different predominant species of bees. However, it is important to note that regions with long winters have a low colony of stingless bees that resulted in being understudied. Biological activities remained one of the most important aspects in the sting and stingless bees' studies and were found to regulate several different cellular pathways. Hence, future studies are critical in promoting and understanding the mechanism of action of sting and stingless bees to act as antimicrobial agents or other potential biological activities.
